# A Student-Led National Conference on Leadership: Broadening the Medical Student Role

**DOI:** 10.15694/mep.2019.000133.1

**Published:** 2019-06-17

**Authors:** Christopher Thomas, Shilpa Mokshagundam, Julia Pitkin, Richard Andresen, Eli Bunzel, Jesse Burk-Rafel, Anna Cassell, Trent Chiang, Laura Derry, Evan Merryman, Skender Najibi, Maria Pliakas, Hanna Saltzman, Kylie Steenbergh, Ammu Vijayakumar, Jeff Wagner, Camille Yongue, Korie Zink, Katie Zurales, Tony Tsai, Michael Dekhtyar, Susan Skochelak, Rajesh Mangrulkar

**Affiliations:** 1 The Brody School of Medicine at East Carolina University; 2 Vanderbilt University School of Medicine; 3 University of Utah School of Medicine; 4 University of Michigan Medical School; 5 Case Western Reserve University School of Medicine; 6 University of North Carolina School of Medicine; 7 Oregon Health & Science University School of Medicine; 8 American Medical Association

**Keywords:** student-led conference, medical student leadership, undergraduate medical education, leadership development, curriculum development, leadership curriculum development, health systems science

## Abstract

This article was migrated. The article was marked as recommended.

Students have traditionally held a singular role in medical education - the learner. This narrow view neglects students unique perspective and ability to shape the future of medical education. In recognizing the need for deliberate leadership skill development and networking opportunities for medical student leaders, the American Medical Association (AMA) supported the first AMA Accelerating Change in Medical Education Student-Led Conference on Leadership in Medical Education. A planning committee of 19 students from seven medical schools collaborated to develop this conference, which took place on August 4-5, 2017 at the University of Michigan, Ann Arbor. The primary goal of the conference was for students to learn about leadership skills, connect with other student leaders, feel empowered to lead change, and continue to lead from their roles as students. Attendees participated in a variety of workshops and presentations focused on developing practical leadership skills. In addition, students formed multi-institutional teams to participate on in the MedEd Impact Challenge, attempting to address issues in medical education such as leadership curriculum development, wellness, and culture change. Post-conference surveys showed an overwhelming majority of students connected with other student leaders, shared ideas, developed collaborations, and felt empowered to enact change. Looking forward, we believe that similar student-led conferences focused on broadening the medical student role would provide avenues for positive change in medical education.

## Problem

Students have traditionally held a singular role in medical education - the learner. This narrow view neglects students’ unique perspective and ability to shape the future of medical education ( Burk-Rafel *et al.,* 2017). While many undergraduate and graduate medical education programs state they are developing the “physician leaders of tomorrow” through deliberate training, leadership development practices are variable and often lack clear objectives or assessment of the skills attained ( Neeley *et al.,* 2017). As a result, students who pursue leadership opportunities within their future careers may do so without having developed a foundational leadership skill set. In addition, opportunities for peer-to-peer student leader networking, an important component of the development of leadership skills, have been limited. In recognizing the need for deliberate leadership skills development and networking opportunities for medical student leaders, the American Medical Association (AMA) supported the first AMA Accelerating Change in Medical Education (AMA-ACE) Student-Led Conference on Leadership in Medical Education.

## Approach

A planning committee of nineteen students from seven medical schools collaborated to plan and develop this conference, which took place on August 4-5, 2017. The committee set the primary goal for the conference: for students to learn about leadership skills, connect with other student leaders, feel empowered to lead change, and continue to lead from their roles as students at their respective medical schools. Students from AMA-ACE Consortium schools ( Appendix 1) submitted proposals for workshops, oral presentations, and poster presentations to share with conference attendees. The planning committee and faculty advisors reviewed and accepted 66 presentations, consisting of 48 poster presentations and 17 oral presentations. The planning committee chose four central themes for the conference: Learn; Connect; Empower; and Impact, as will be further defined and described below.

## Learn

Students first engaged in a workshop on the Competing Values Framework leadership assessment ( Cameron *et al.,* 2014). After determining their leadership styles, students were encouraged to form teams of five students with different leadership styles. Teams attended workshops designed and led by students, which provided an engaging experience in developing communication, team building, and problem solving skills. Attendees participated in a mock debate regarding current topics in medical education, identifying potential individuals of influence and refining communication skills by using common tactics of persuasion. Students learned team-building skills through the Tuckman Model of Group Development( Tuckman, 1965), which emphasizes the “forming-storming-norming-performing” phases that allow a team to plan and solve problems. Students then applied these lessons through interactive small group activities. Students developed problem solving skills by applying Plan-Do-Study-Act cyclesand critical thinking processes to self-identified challenges related to their educational experience ( Gillam *et al.,* 2013).

Dr. Alisha Moreland-Capuia, MD, executive director for the Avel Gordly Center for Healing, assistant professor of psychiatry at the Oregon Health and Science University School of Medicine, and Co-Founder of The Capuia Foundation gave a keynote presentation entitled “Learning to Lead From Where You Are.” Through this presentation, attendees learned about approaches and strategies to act as leaders from their current positions, and the speaker charged attendees to continue leading throughout their medical school careers and beyond.

## Connect

Presenters shared student-led innovations and initiatives in medical education, highlighting successes and challenges of student leadership. Through workshops and presentations, students connected with peers from across the country with shared interests in leadership in medical education, helping to develop a personal network critical to a career in academic medicine. These presentations and workshops allowed students to disseminate projects from their home institutions such as women leadership development programs, health policy education programs, and mass casualty trauma simulations. The planning committee selected submissions to provide attendees with ideas to connect with student leaders and implement similar initiatives at their home institutions.

## Empower

Following the keynote presentation and student-led workshops and presentations, the teams of students participated in the MedEd Impact Challenge, a central activity thread through the conference. Attendees used their skills and ingenuity to develop potential solutions to three pressing issues in medical education, which revolved around leadership curriculum development, wellness, and culture change ( Appendix 2). Students were encouraged to work out of their comfort zone in developing detailed solutions to the proposed problems by working with their teammates of different leadership styles. Following the topic reveal, teams were given two hours to develop their innovative approach to one of the MedEd Impact Challenge questions. Students utilized the skills recently gained from conference workshops, such as Plan-Do-Study-Act cycles, and submitted a written proposal. A panel of faculty judges from the ACE Consortium reviewed these written proposals and nine of fourteen teams were chosen as finalists. Finalists then refined their innovation and pitched it to all conference attendees. A winning team was chosen for each of the MedEd Impact Challenge questions, and each team was awarded the opportunity to present their solution at the AMA ChangeMedEd Conference in Chicago, IL on September 15, 2017.

## Impact

All MedEd Impact Challenge proposals were shared with the principal investigators from the AMA ACE Consortium schools in order to continue dialogue and empower student leaders at these institutions. Students were also encouraged to influence medical education at their home institutions by implementing ideas, programs, and curricula from the leadership conference. The goal was for students to refine their skills in order to serve as agents of change in medical education and to challenge the traditional “student as learner” narrative.

## Outcomes

In total, 105 students representing all 32 medical schools in the AMA-ACE Consortium attended the conference. A post-conference evaluation survey was sent to all conference attendees; 24 of 105 students responded (23% response rate). Over 95% of respondents (n=22) rated the conference overall as “Good or Excellent” ( Appendix 3). Greater than 90% of respondents stated they felt connected with other leaders, shared ideas, and developed collaborations, as well as empowered and energized to enact positive change in medicine. The majority of students agreed the overarching goals of the conference (Learn, Connect, Empower, and Impact) were met. Post-conference free response surveys stated the conference “raised my level of engagement and passion for making changes in medical education” and “challenged students to critically think and discuss issues pertinent in healthcare and medical school today and develop a viable solution.” Student responses also stated the conference was “very engaging” and “empowering.” Respondents stated they enjoyed working with engaged and highly motivated students from other medical schools and were inspired by conference attendees. We hope that the conference bolstered students’ leadership skill sets, empowered them to lead change at their home institutions, and emboldened them to assume leadership roles in their careers.

In addition to the valuable skills learned from attending the conference, students on the planning committee developed leadership skills through the organization and execution of the conference programming and evaluation, and formed a lasting peer leader network.

## Take Home Messages

Conference attendees were encouraged to continue their involvement in medical education as they progress through their careers. After bringing together students from across the country for networking, skills building, and brainstorming, conference attendees were charged to return to their home institutions and share the skills and knowledge they gained. As the first example of a student-led national conference on medical education leadership, more robust evaluation is needed, including additional conferences and going beyond measures of satisfaction to assess skills, knowledge, and behaviors acquired. The long-term impact of this type of conference could be measured by surveying participants at delayed intervals, and interviewing the Principal Investigators at the consortium schools to determine how the student attendees have influenced change in medical education in the year since the conference.

Given the success of this initiative, we anticipate that similar conferences will be held in the future, including even more medical student leaders. We hope this initiative will form a network of students within the national AMA-ACE Consortium that will catalyze changes in medical education and foster student medical education leaders and potential future leaders in medicine.

## Notes On Contributors

The above authors were either members or advisors of the American Medical Association (AMA) Student-Led Leadership Conference Planning Committee. This Committee was comprised of students and faculty advisors from seven medical schools within the AMA Accelerating Change in Medical Education Consortium.

## Appendices

### Appendix 1. American Medical Association Accelerating Change in Medical Education Consortium

A.T. Still University, School of Osteopathic Medicine in Arizona

Case Western Reserve University School of Medicine*

CUNY School of Medicine

Dell Medical School at the University of Texas at Austin

Eastern Virginia Medical School

Emory University School of Medicine

Florida International University Herbert Wertheim College of Medicine

Harvard Medical School

Indiana University School of Medicine

Mayo Clinic School of Medicine

Michigan State University College of Osteopathic Medicine

Morehouse School of Medicine

New York University School of Medicine

Ohio University Heritage College of Osteopathic Medicine

Oregon Health & Science University School of Medicine*

Pennsylvania State University College of Medicine

Rutgers Robert Wood Johnson Medical School

Sidney Kimmel Medical College at Thomas Jefferson University

The Brody School of Medicine at East Carolina University*

The University of Chicago Pritzker School of Medicine

The Warren Alpert Medical School of Brown University

University of California, Davis, School of Medicine

University of California, San Francisco, School of Medicine

University of Conecticut School of Medicine

University of Michigan Medical School*

University of Nebraska Medical Center College of Medicine

University of North Carolina School of Medicine*

University of North Dakota School of Medicine and Health Sciences

University of Texas Rio Grande Valley School of Medicine

University of Utah School of Medicine*

University of Washington School of Medicine

Vanderbilt University School of Medicine*

*Schools represented on the AMA Accelerating Change in Medical Education Student-Led Conference on Leadership Planning Committee

### Appendix 2. MedEd Impact Challenge Questions

1. How can medical schools help students to both build leadership skills and prioritize leadership as a critical component of personal and professional growth?

2. How can we create a culture in which students are empowered to be change agents in highly stratified organizational structures?

3. How can medical schools contribute to efforts to prevent student burnout and create environments in which students flourish in the midst of stress and intensity?

### Appendix 3. Responses from Post-Conference Survey

**Table:**

Outcome	Response
Overall conference rating - Good or Excellent	95.7% (22/23)
Learn: I learned about leadership in medical education - Agree or Strongly Agree	83.3% (20/24)
Connect: I connected with other leaders, shared ideas, and developed collaborations - Agree or Strongly Agree	91.7% (22/24)
Empower: I was empowered and energized to enact positive change in medicine - Agree or Strongly Agree	91.7% (22/24)
Impact: I developed knowledge, skills, and behaviors for impacting medical education and healthcare - Agree or Strongly Agree	79.2% (19/24)

## References

[ref1] Burk-Rafel J. Jones R.L. and Farlow J.L. (2017) Engaging learners to advance medical education. Academic Medicine. 92(4), pp.437–440. 10.1097/acm.0000000000001602 28198724

[ref2] Cameron K.S. Degraff J. Quinn R.E. and Thakor A.V. (2014) Competing Values Leadership Creating Value in Organization. Northamptom: Edward Elgar Publishing.

[ref3] Gillam S. and Siriwardena N. (2013) Frameworks for improvement: clinical audit, the plan-do-study-act cycle and significant event audit. Quality in Primary Care. 21(2), pp.123–130. 23735693

[ref4] Neeley S.M. Clyne B. and Resnick-Ault D. (2017) The state of leadership education in US medical schools: results of a national survey. Medical Education Online. 22(1), p.1301697. 10.1080/10872981.2017.1301697 28298155 PMC5419299

[ref5] Tuckman B.W. (1965) Developmental sequence in small groups. Psychological Bulletin. 63(6), pp.384–399. 10.1037/h0022100 14314073

