# Supplementary material for: A Student-Led National Conference on Leadership: Broadening the Medical Student Role
Source: MedEdPublish (2016). 2019 Jun 17;8:133. [Version 1] doi: 10.15694/mep.2019.000133.1 (PMC10712505; doi:10.15694/mep.2019.000133.1)
Supplement: Peer review history

# Reviewer report: Reviewer response for version 1

**DOI:** 10.21956/mep.19814.r29838

**Published:** 2019-07-30

**Authors:** Jayne Garner

**Affiliations:** 1 university of liverpool

This review has been migrated. The reviewer awarded 4 stars out of 5 Interesting paper providing insight into the students approach and focus upon leadership. The range of topics covered appears to be vast and this descriptive account gives a brief overview. More detailed information on how students were selected, their demographics and robust evaluation of the sessions would enable further understanding of the conference and its long term impact.

Reviewer Expertise:

NA

No decision status is available

# Reviewer report: Reviewer response for version 1

**DOI:** 10.21956/mep.19814.r29839

**Published:** 2019-06-26

**Authors:** Judy McKimm

**Affiliations:** 1 Swansea University

This review has been migrated. The reviewer awarded 3 stars out of 5 I was very interested to read about this conference and the associated activities. The main strength of this is the networking and ongoing activities and it will be interesting to see how sustainable this model is. I wondered what leadership theories/approaches underpinned this and would be keen to see a deeper academic reflection on the various approaches role modeled and foregrounded.

Reviewer Expertise:

NA

No decision status is available

# Reviewer report: Reviewer response for version 1

**DOI:** 10.21956/mep.19814.r29836

**Published:** 2019-06-25

**Authors:** BALAJI ARUMUGAM

**Affiliations:** 1 TAGORE MEDICAL COLLEGE AND HOSPITAL

This review has been migrated. The reviewer awarded 4 stars out of 5 This is the fantastic effort by the students which showed that they are really true leaders of future. As a reviewer and a medical education team member in my institute, I am very happy and excited to read an article on student led conference focusing on leadership. The entire planning and execution of the conference was structured by the students and the outcome, feedback of the participants suggests that this conference would have been a great success. Student led planning committee of nineteen students conducted a conference to learn about leadership skills focusing on central themes like, learn, connect, empower and the impact was well executed overall. Congrats to the entire team. I would like to know more details on this conference so that I can plan such an event in our institution.

Reviewer Expertise:

NA

No decision status is available

# Reviewer report: Reviewer response for version 1

**DOI:** 10.21956/mep.19814.r29837

**Published:** 2019-06-19

**Authors:** Ariela Marshall

**Affiliations:** 1 Mayo Clinic

This review has been migrated. The reviewer awarded 5 stars out of 5 I was extremely impressed by this manuscript. The paper was incredibly well-written and the content was fascinating and inspiring. The idea of getting medical students involved as leaders from an early stage in training is excellent. Most physicians will step into leadership roles at some point during their careers, and teaching students the leadership skills they will need as part of medical training seems not only reasonable but in fact like something that should be mandatory. As we develop new educational strategies for the Millennial generation and beyond, we must realize that medicine will no longer be a “vertical” hierarchy but instead a “horizontal” playing field where learners at all stages can grow by learning from each other. Teaching students leadership skills and then asking them to use these skills to problem-solve (the “MedEd Impact Challenge”) is a wonderful way to involve learners even at the beginning stages. I was very excited to read this paper and hope that you publish it soon so that it can be disseminated to leaders in medical schools throughout the country!

Reviewer Expertise:

NA

No decision status is available
